# Good outcome with conservative treatment of delayed spinal epidural hematoma following combined spinal-epidural anesthesia: a rare case report

**DOI:** 10.1186/s12871-024-02619-1

**Published:** 2024-07-12

**Authors:** Hui Yao, Xuejie Li, Shize Leng, Hui Zhang

**Affiliations:** 1 Department of Anesthesiology, The Affiliated Guangdong Second Provincial General Hospital of Jinan University, No. 466, Shi liugang road, Hai zhu District, Guangzhou, Guangdong Province China; 2 Department of Anesthesiology, West China Hospital, Sichuan University, Chengdu, Sichuan Province China

**Keywords:** Delayed spinal epidural hematoma, Central neuraxial block, Combined spinal-epidural anesthesia, Low molecular weight heparin, Conservative treatment, Dynamic neurological evaluation

## Abstract

**Background:**

Delayed spinal epidural hematoma (SEH) following central neuraxial block (CNB) is a rare but serious complication. The underlying causes of SEH associated with neuraxial anesthesia are still unclear. Furthermore, the decision between surgical intervention and conservative management for SEH remains a complex and unresolved issue.

**Case Presentation:**

We report a case of delayed SEH in a 73-year-old woman who underwent vaginal hysterectomy under combined spinal-epidural anesthesia, with the administration of postoperative anticoagulants to prevent deep vein thrombosis on the 1st postoperative day (POD). She experienced symptoms 56 h after CNB. Magnetic resonance imaging (MRI) revealed a dorsal SEH at the L1-L4 level with compression of the thecal sac. On conservative treatment, full recovery was achieved after six months.

**Conclusions:**

This case reminds anesthesiologists should be alert to the possible occurrence of a delayed SEH following CNB, particularly with the administration of anticoagulants. Immediate neurological evaluation of neurological deficit and MRI are advised. Conservative treatment combined with close and dynamic neurological function monitoring may be feasible for patients with mild or nonprogressive symptoms even spontaneous recovery.

## Background

The incidence of spinal epidural hematoma (SEH) associated with central neuraxial block (CNB) is extremely low, estimated to range from 1:150,000 to 1:220.000 [1, 2]. However, if the SEH is not diagnosed timely and treated appropriately, it can cause potentially severe neurological loss or even paraplegia. It has been reported in the literature that acute SEH is more common, usually occurring within 24 h post-surgery, and exceeding 72 h is defined as delayed SEH [3]. The exact etiology of SEH associated with neuraxial anesthesia remains unclear. The risk factors reported in the literature mainly include advanced age, pregnancy, hypertension, history of spinal fracture and spinal cord injury, perioperative anticoagulation therapy, coagulation dysfunction, spinal canal internal deformity, puncture needle size, multiple punctures, and difficult catheter placement [2].

We report a case of delayed SEH following combined spinal-epidural anesthesia (CSEA). The originality of our report arises in three points: The case involved the simple lumbar radicular symptoms. The SEH was solved spontaneously. On conservative treatment, full recovery was achieved.

## Case presentation

We present the case of a 73-year-old female patient, who provided written and signed consent for the publication of this report, with a BMI of 18.6 (height 150 cm and weight 42 kg) and American Society of Anesthesiologists Physical Status of II. Her medical history includes hypertension and T8 percutaneous vertebroplasty. She exhibited no spinal tenderness and the muscle strength of all four limbs was grade 5. Her laboratory investigations, including platelet count, coagulation parameters (prothrombin time, international normalized ratio, and activated partial thromboplastin time) and renal function, were within normal limits. Spiral CT scan revealed a thoracic vertebral compression fracture and lumbar disc herniation at L4/5. Vaginal hysterectomy and vaginal wall repair were planned.

Before anesthetics administration, the patient’s vital signs were monitored: non-invasive blood pressure was 142/87mmHg, heart rate was 91 beats/min, and oxygen saturation was 97%. She received combined spinal-epidural anesthesia (CSEA) by an experienced anesthesiologist with 20 years of experience. The 16G epidural puncture needle was successfully punctured into the epidural space at the L3/4 midline in the first attempt without a bloody tap, subsequently, the 25G lumbar puncture needle was used to penetrate into the subarachnoid space. No paresthesia was elicited, clear cerebrospinal fluid was obtained and 12 mg of 0.5% ropivacaine was administered intrathecally. An epidural catheter was placed. After the sensory block was at level T8, surgery commenced and completed successfully within two hours. The epidural catheter was removed immediately after surgery, and the block was fully reversed six hours later.

She was able to stand and walk independently, with normal muscle strength and sensation in all four limbs in the morning on the 1st postoperative day (POD) when the anesthesiologist conducted a routine postoperative follow-up. In the evening of 1st POD, given the patient’s advanced age and the two-hour duration of the surgery, subcutaneous low-molecular-weight heparin (4100 anti-factor Xa IU) was administered to prevent deep vein thrombosis (DVT). However, in the afternoon on the 2nd POD, more precisely, 56 h after CNB, she experienced sudden-onset severe and sharp low-back pain after stumbling while walking, which was followed by muscle weakness in her left leg, rendering her unable to lie down, walk, or even stand. The muscle strength of the left lower limb was grade as 2/5. Six hours later, the neurosurgeon consulted and performed a physical examination, which revealed which revealed noticeable tenderness of left lumbar paravertebral area, hypesthesia of lateral left thigh and calf, muscle weakness of the left lower limb (muscle strength rated as 2/5 for hip flexion, 3/5 for knee extension and 4/5 for plantar flexion), absence of patellar tendon and Achilles tendon reflexes. Fortunately, there were no signs of urinary dysfunction or saddle sensory disturbances. There were strikingly noticeable ecchymoses present on her abdomen and limbs. The neurosurgeon suggested that a magnetic resonance imaging (MRI) of the lumbar spine should be performed immediately. However, MRI couldn’t be performed due to significantly worsened back and left leg pain when she was lying flat, despite the administration of tramadol and NSAIDs. In a semi-recumbent position, however, the pain was relieved and became tolerable for her. Therefore, neurotrophic treatment was administered, including reducing nerve edema with mannitol, anti-inflammatory medication such as methylprednisolone, neurotrophic treatment with mecobalamin, pain control with NSAIDs. Fortunately, she experienced significant pain relief and was able to stand unassisted and lie down 4 days after symptoms.

**Fig. 1:**
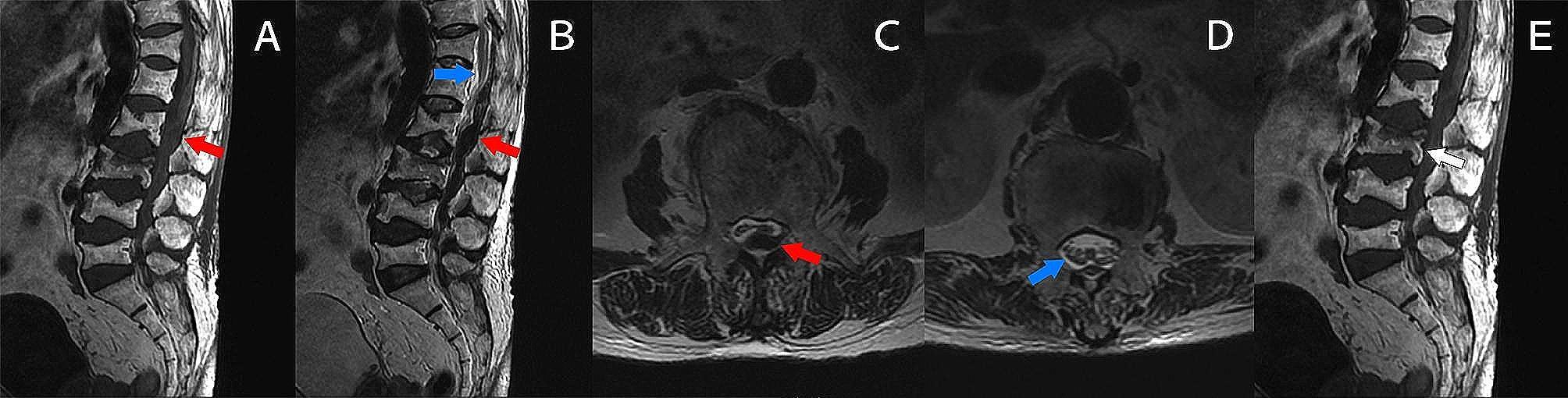
Initial MRI performed five days after symptoms onset revealed a dorsal SEH at the level of L1-L4 with compression of the thecal sac (red arrow) (**A-C**) but not the conus medullaris (blue arrow)(**D**). The SEH displayed a mixed signal with isointense and slightly hypointense to the spinal cord on T1WI sagittal image(**A**) and hypointense on T2WI sagittal image (**B**) and T2WI axial image showing(**C**). Additionally, the images revealed a recent L3 vertebral compression fracture (white arrow) (**E**)

Lumbar MRI, performed approximately 5 days after symptoms onset, revealed a dorsal SEH at L1-L4 level, predominantly on the left side, with a maximum transverse diameter of 8 mm, with compression of thecal sac and the L2-5 nerve roots, rather than conus medullaris. There was also a fresh osteoporotic vertebral compression fracture (OVCF) at lumbar (L3) and disc herniation of the L4-5 without compression of nerve roots (Fig. 1).

**Fig. 2:**
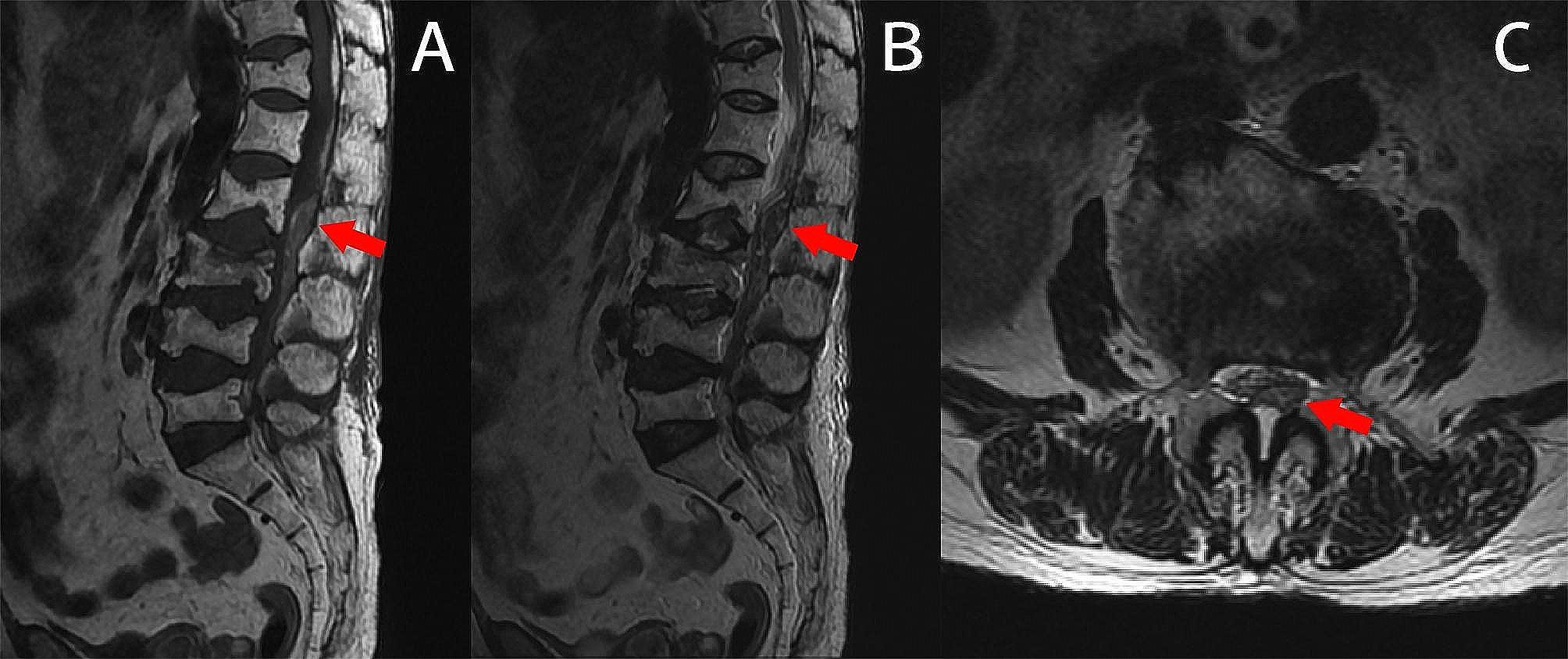
Follow-up MRI four days later revealed that the SEH (red arrow) had decreased in size (**A-C**). SEH: spinal epidural hematoma. MRI: magnetic resonance imaging. WI: weighted imaging

After immediate multidisciplinary consultation, conservative treatment was advised for SEH based on the following factors. Firstly, the patient presented with a neurologic status of D, as defined by the American Spinal Injury Association Impairment Scale (ASIA) score, and exhibited spontaneous improvement in neurological deficit. Secondly, the patient mainly presented with compression symptoms of nerve root, rather than spinal cord or cauda equina. Thirdly, laminectomy involving four lumbar segments poses considerable risks due to the lengthy procedure time and significant associated trauma, especially given the patient’s advanced age. Finally, after thoroughly discussing the risks and benefits with the patient, she chose conservative treatment. Additionally, conservative treatment was chosen for the L3 osteoporotic vertebral compression fracture because the fracture was stable and didn’t compress the spinal cord and nerve roots. Conservative treatment included the cessation of anticoagulants, continuation of previous medical treatments, and rigorous monitoring of neurological function recovery. Subsequent MRI scan, taken four days later, revealed a reduction in the size of the hematoma (Fig. 2). Under conservative treatment, she reported no pain and further improvement of the weakness of her left lower limb, and was able to walk with assistance approximately 15 days after the onset of symptoms. She was discharged 19 days POD and achieved unassisted walking six months later during follow-up.

## Discussion and conclusions

A SEH may be either spontaneous or secondary to trauma, coagulation disorders, or regional anesthetic techniques. SEH, described following neuraxial anesthesia, is more common after epidural than spinal anesthesia and is usually seen in conjunction with coagulation abnormalities or administration of anticoagulants [4]. In the present case, multifactorial etiology of SEH is more likely. Firstly, inadvertent trauma to the epidural veins during CSEA cannot be ruled out completely, although the procedure was done successfully in first attempt by an experienced anesthesiologist and the symptoms appeared 56 h after CNB. Further, anticoagulation increased the risk of developing delayed SEH for the patient. The doses of drugs for prophylactic anticoagulants were relatively large (low-molecular-weight heparin 4100 anti-factor Xa IU one per day) in consideration of the patient’s weight (42 kg), and the time of anticoagulant therapy was too long, which prolonged until one week POD [5]. The patient’s ecchymoses on her abdomen and limbs disappeared 10 days after surgery. Thirdly, new-onset lumbar 3 vertebral compression fracture may result in shearing or tearing of the epidural veins, while the anticoagulation therapy likely further contributed to the hemorrhage [6]. Finally, hypertension may be another etiology.

Symptomatic epidural hematoma is an accumulation of blood in the spinal canal that compresses the thecal sac, cauda equina, or spinal cord, leading to neurologic dysfunction. The most common clinical presentations are new back pain, sensory and motor deficit (68%) or even paraplegia, and the typical symptoms are cauda equina syndrome (8%), but radicular pain is uncommon. Groen et.al [7] reported that 9% of a series of 64 SSEH cases treated conservatively presented an isolated radicular compromise. In the present case, her primary clinical symptoms included low-back pain, radicular pain and motor deficit in the left lower limb, without any signs of sphincter dysfunction or saddle sensory disturbances. These findings suggest that the SEH compressed the left L2-5 nerve roots rather than the cunus medullaris or cauda equina, which is consistent with the MRI images. Compression of nerve roots may be due to the closure of the intervertebral foramina in elderly patients, leading to local compression and neurological symptoms. One review [8] of 229 SEH cases showed the median to first symptoms was 24 h (5–48 h), after the predisposing event (i.e. CNB or removal of catheter). In our case, the symptoms appeared 56 h after CNB.

A SEH can have catastrophic complications. So, MRI, which is the best diagnostic technique, should be performed immediately to confirm the diagnosis in cases of suspected SEH so as not to miss the optimal treatment opportunity. Unfortunately, in the present case, MRI couldn’t be performed timely because of her inability to lying down. In fact, we could have administered potent opioids to alleviate pain so that MRI could be performed earlier. Moreover, when persistent neurological deterioration is considered, early surgical decompression is an effective treatment modality, rather than imaging findings, as the sole criterion for a treatment strategy [9]. Fortunately, the patient’s neurological function recovery was good.

Once the diagnosis of an SEH has been established, immediate neurosurgical consultation is very important to evaluate neurological deficits and making management decisions. The decision between conservative and surgical treatment often largely depends on several factors, including the size and location of the SEH, the severity and progression of symptoms and the patient’s overall health and medical history. A multicenter retrospective study suggest that timely surgical decompression is recommended for patients with moderate/severe neurologic deficits or progressive neurologic deterioration [10]. In addition, urgent evaluation and surgical intervention are recommended in most cases of compressive cauda equina syndrome and spinal cord compression [11].

While surgical decompression within 12 h of onset is generally associated with favorable neurological outcomes [12], there is growing evidence supporting conservative management, Firstly, as reported in studies, conservative treatment may be feasible in patients with mild and nonprogressive neurologic impairment, even spontaneous recovery despite the significant size of SEH [8, 13, 14]. Siasios ID et.al [9] reported the patient’s recovery with conservative management for a SEH after epidural anesthesia. Raasck K et.al [15] suggested that patients with an initial ASIA score of D are more likely to have a good prognosis with conservative treatment. 33% of patients managed conservatively had an initial score of A or B, all improving to a score of D or E without surgery. For cases with paralysis recovery start timey within 11 h from onset, non-operative observation is applicable and result in good outcomes [13]. Secondly, a conservative approach may lead to a complete recovery and may be considered as a good option in the case of radicular involvement [16]. In my opinion, several factors contribute to this. The nerve roots are protected and cushioned by the surrounding structures as they pass through the foramen. The blood supply to the nerve roots is relatively abundant. The symptoms are primarily localized and milder when the SEH compresses the nerve roots, making it more responsive to conservative treatment and resulting in a better prognosis. In our case, the patient had an initial score of D and spontaneous early improvement of neurological function. She primarily presented with symptoms of radicular compression rather than spinal cord or cauda equina. Laminectomy presents considerable risks, given the procedure’s extended duration and significant associated trauma, along with the patient’s advanced age. Thirdly, laminectomy involving four lumbar segments poses considerable risks. Finally, the patient chose conservative treatment. Considering these factors, conservative treatment was deemed appropriate for this patient.

However, it is critical to clearly communicate with the patient that conservative treatment may not guarantee complete neurological recovery and there could be further deterioration of neurological function during the treatment. Additionally, close and dynamic neurological function monitoring during conservative treatment, is of utmost importance. Should any changes arise, timely neurological consultation should be sought, and the possibility of surgical intervention considered if necessary.

The ultimate neurological outcome is influenced by several factors: the extent of neurological deficits, the operative interval [17], the rate of hematoma progression, the extent of neurological deficits, and the size of the hematoma [1]. The favorable prognosis of the patient can be attributed to the small size of the SEH, mild neurological deficits and symptom improvement following conservative treatment, and diligent monitoring of neurological function.

We have to admit that the limitation of our case is that we didn’t conduct an immediate MRI scan because of the patient’s severe low back pain, which prevented her from lying down. In fact, we could have administered potent opioids to alleviate pain so that MRI could be performed earlier. Fortunately, the timely application of conservative treatment led to the patient’s successful recovery.

In conclusion, the potential for delayed SEH following CNB, particularly with the administration of anticoagulants, must always be taken into consideration. It is crucial to monitor neurological function promptly after surgery and to seek immediate neurological evaluation and MRI if abnormalities are detected. Once a SEH is diagnosed, the decision between conservative and surgical treatment should be based on a comprehensive evaluation of the patient’s condition, and the risks and benefits of them should be balanced. Conservative treatment may generally appropriate for patients with mild, nonprogressive symptoms, even gradual improvement, and who do not exhibit signs of cauda equina syndrome or spinal cord compression. However, it is of utmost importance to thoroughly discuss the risks of conservative treatment with the patient and close and dynamic neurological function monitoring during conservative treatment, so that surgical intervention can be promptly carried out if deterioration occurs.

## Data Availability

No datasets were generated or analysed during the current study.

## Abbreviations

SEH: Spinal epidural hematoma
CNB: Central neuraxial block
CSEA: Combined spinal-epidural anesthesia
POD: Postoperative day
MRI: Magnetic resonance imaging
DVT: Deep vein thrombosis
NSAIDs: Non-steroidal anti-inflammatory drugs
BMI: Body mass index
